# Neuroprotective Effects of Pinostrobin in a Rat Model of Kainic Acid-Induced Epilepsy

**DOI:** 10.3390/ijms27198579

**Published:** 2026-09-25

**Authors:** Jinatta Jittiwat, Ratchaniporn Kongsui, Tichanon Promsrisuk, Napatr Sriraksa, Sitthisak Thongrong

**Affiliations:** 1 Faculty of Medicine, Mahasarakham University, Mahasarakham 44000, Thailand; jinatta.j@msu.ac.th; 2 Division of Physiology, School of Medical Sciences, University of Phayao, Phayao 56000, Thailand; ratchaniporn.ko@up.ac.th (R.K.); tichanon.pr@up.ac.th (T.P.); napatr.sr@up.ac.th (N.S.); 3 Division of Anatomy, School of Medical Sciences, University of Phayao, Phayao 56000, Thailand

**Keywords:** pinostrobin, epilepsy, Bcl-2-associated X protein (Bax), B-cell lymphoma 2 (Bcl-2), caspase-3, apoptosis

## Abstract

Epilepsy is a serious neurological disorder characterized by recurrent seizures and neuronal loss. Prolonged seizure activity is associated with changes in apoptosis-related proteins, including Bcl-2-associated X protein (Bax) and caspase-3, as well as alterations in microglial and astroglial responses. This study investigated the neuroprotective effects of pinostrobin, a bioflavonoid, in a kainic acid (KA)-induced epilepsy model and examined its effects on apoptosis-related proteins. Pinostrobin (40 mg/kg) was administered orally once daily for 7 days before and 14 days after intracerebroventricular injection of KA. KA injection resulted in neuronal cell loss, increased number of ionized calcium-binding adapter molecule 1 (Iba1)-positive microglia, and reactive astrocytosis in the cornu ammonis 1 (CA1), cornu ammonis 3 (CA3), and hilar regions. These changes were accompanied by decreased levels of protein kinase B (Akt), phosphorylated-Akt (p-Akt), and the anti-apoptotic protein B-cell lymphoma 2 (Bcl-2), together with increased levels of Bax and caspase-3. Pinostrobin treatment significantly attenuated neuronal cell loss, reduced the number of Iba1-positive microglia, and increased glial fibrillary acidic protein (GFAP) immunoreactivity in the CA1, CA3, and hilar regions. Moreover, pinostrobin increased Akt, p-Akt, and Bcl-2 levels while decreasing Bax and caspase-3 levels. Collectively, these findings suggest that pinostrobin has potential neuroprotective effects in the KA-induced epilepsy model and is associated with changes in apoptosis-related proteins and glial responses.

## 1. Introduction

Epilepsy ranks among the most serious neurological disorders and is characterized by recurrent, spontaneous seizures, affecting approximately 70 million people worldwide [1]. Kainic acid (KA), a glutamate analog, is widely used to induce an excitotoxic model of epilepsy. Administration of KA causes overstimulation of kainate and α-amino-3-hydroxy-5-methyl-4-isoxazolepropionic acid (AMPA) receptors, resulting in a large influx of calcium ions (Ca^2+^) into neurons. This increase in Ca^2+^ levels causes mitochondrial dysfunction and endoplasmic reticulum stress, subsequently triggering apoptotic pathways that contribute to hippocampal neuronal death and the development of epileptic seizures [2,3]. KA-induced seizures result in decreased levels of B-cell lymphoma 2 (Bcl-2), an anti-apoptotic protein, and increased levels of Bcl-2 associated X (Bax), a pro-apoptotic protein; this shift leads to mitochondrial leakage and subsequent activation of caspase-3, ultimately inducing neuronal death [2,4,5,6]. Additionally, KA-induced Ca^2+^ overload is associated with glial activation, characterized by the clustering of reactive microglia and astrocytes within the hippocampus [7]. Reactive microglia release pro-inflammatory cytokines, such as tumor necrosis factor-α (TNF- α) and interleukin-1β (IL-1β), and activate apoptotic enzymes including caspase-3 and caspase-7, leading to neuronal apoptosis [8]. Therefore, natural compounds capable of suppressing microglial responses and the subsequent release of pro-inflammatory cytokines and apoptotic enzymes represent a promising strategy for attenuating KA-induced neurotoxicity.

Pinostrobin is a natural flavonoid with several pharmacological properties including antioxidant, anti-inflammatory, and neuroprotective activities [9,10]. Research has shown that pinostrobin protects against beta-amyloid-induced neurotoxicity in an Alzheimer’s disease model by reducing intracellular Ca^2+^ influx, stabilizing mitochondrial function, and lowering the Bax/Bcl-2 ratio, thereby preventing neuronal apoptosis [11]. Pinostrobin has also been reported to exert neuroprotective effects in neurotoxin-induced animal models of Parkinson’s disease through upregulation of the phosphoinositide 3-kinase/protein kinase B (PI3K/Akt) signaling pathway [12]. These neuroprotective effects have been attributed to the ability of flavonoids such as pinocembrin and pinostrobin to cross the blood–brain barrier (BBB) [13]. In addition, pinostrobin has been reported to have a favorable safety and toxicity profile. A previous study in male Wistar rats found that a single oral administration of pinostrobin at a high dose of 500 mg/kg resulted in no mortality. Furthermore, this treatment caused no significant changes in body weight, vital organ weights, or blood biochemical parameters compared with the control group, supporting its safety for in vivo applications [14].

Although pinostrobin has demonstrated neuroprotective effects by reducing apoptotic markers and upregulating survival pathways, its effects on KA-induced neuronal degeneration remain unclear. Therefore, this study aimed to investigate the potential neuroprotective effects of pinostrobin on KA-induced neuronal cell loss and its effects on apoptosis-related proteins and glial responses in the hippocampus.

## 2. Results

### 2.1. Pinostrobin Protects Against KA-Induced Neuronal Cell Loss in the Hippocampus

KA-induced seizures result in extensive neuronal loss in the CA1 and CA3 regions [5,15]. In this study, neuronal cell survival was assessed by Nissl staining following intracerebroventricular (i.c.v.) injection of KA (Figure 1A–I). The number of pyramidal neurons was significantly decreased in the cornu ammonis 1 (CA1) and cornu ammonis 3 (CA3) regions of the KA group compared with that of the control animals (*p* < 0.001). Notably, the pinostrobin-treated group exhibited a significantly higher numbers of pyramidal neurons in the CA1 (*p* < 0.01) and CA3 (*p* < 0.001) than the KA group (Figure 1J-K).

Hilar neurons play a crucial role in regulating neuronal excitability, and their loss contributes to the development of epileptic seizures [16,17]. Fourteen days after KA injection, the number of hilar neurons was significantly lower in the KA group than in control animals (*p* < 0.05). However, the pinostrobin-treated group showed significantly less loss of hilar neurons than the KA-alone group (*p* < 0.05) (Figure 1L). These findings indicate that pinostrobin exerts a neuroprotective effect against KA-induced hippocampal neuronal loss.

### 2.2. Pinostrobin Enhances Reactive Astrocytosis in the Hippocampus Following KA-Induced Neuronal Cell Loss

Reactive astrocytosis refers to the activation of astrocytes in response to brain injury and status epilepticus. Astrocytes play a crucial role in glutamate homeostasis, and astrocytic dysfunction may therefore contribute to excitotoxic brain damage. Evidence suggests that enhanced astrocytosis is associated with the preservation of hippocampal neurons and the maintenance of gamma-aminobutyric acid (GABA) levels in response to KA-induced neuroinflammation [18]. In the current study, KA administration induced extensive astrocytosis with increased glial fibrillary acidic protein (GFAP) immunoreactivity throughout the hippocampus compared with the control group (Figure 2A–I). Pinostrobin treatment further significantly increased GFAP immunoreactivity in the CA1, CA3, and hilus compared with the KA-alone group (*p* < 0.05) (Figure 2J–L). These findings suggest that enhanced reactive astrocytosis may contribute to the neuroprotective effects of pinostrobin following KA-induced seizures.

### 2.3. Pinostrobin Reduces the Number of Iba1-Positive Microglia in the Hippocampus Following KA Administration

KA-induced seizures trigger marked proliferation of microglia within the hippocampus. Upon activation, microglia undergo morphological changes characterised by shortening and thickening of cellular processes and enlargement of the cell body, resulting in an amoeboid morphology [19]. Chronic microglial activation leads to the release of pro-inflammatory cytokines such as tumour necrosis factor-α and interleukin-1β, which contribute to hippocampal neuronal loss and astrocyte dysfunction [20]. Thus, preventing microglia-mediated neuroinflammation may protect against hippocampal neurodegeneration following KA injection [21]. To examine whether pinostrobin affects the microglial response to KA-induced seizures, ionised calcium-binding adaptor molecule 1 (Iba1) immunohistochemical staining was used to detect microglia. Fourteen days after KA injection, KA-treated animals displayed a significant increase in the number of Iba1-positive microglia throughout the hippocampus compared with control animals (Figure 3A–I). However, quantitative analysis showed that pinostrobin treatment significantly decreased the number of Iba1-positive microglia in the CA1 (*p* < 0.05), CA3 (*p* < 0.01), and hilus (*p* < 0.01) compared with the KA-alone group (Figure 3J–L). These results indicate that pinostrobin suppresses the microglial response and may thereby protect against hippocampal neuronal loss following KA-induced seizures.

### 2.4. Pinostrobin Decreases the Bax/Bcl-2 Ration in KA-Induced Seizures

To examine the potential neuroprotective effects of pinostrobin in KA-induced seizures, the hippocampal protein levels of Bax and Bcl-2 were evaluated by Western blotting. KA administration resulted in increased levels of the pro-apoptotic protein Bax and decreased levels of the anti-apoptotic protein Bcl-2, changes associated with seizure activity and hippocampal cell death [5]. The Western blot shown in Figure 4A illustrates the protein expression levels of Bax and Bcl-2. In the present study, KA injection increased Bax expression and the Bax/Bcl-2 ratio (Figure 4B,D) while decreasing Bcl-2 expression (Figure 4C). Bax expression and the Bax/Bcl-2 ratio were significantly increased in the KA-alone group compared with the control group (*p* < 0.05 and *p* < 0.001, respectively) (Figure 4B,D). Conversely, pinostrobin treatment significantly decreased Bax expression (*p* < 0.05) and the Bax/Bcl-2 ratio (*p* < 0.001) compared with the KA-alone group. Pinostrobin also significantly increased Bcl-2 expression compared with the KA-alone group (*p* < 0.05) (Figure 4C).

### 2.5. Pinostrobin Attenuates Caspase-3 Activation but Upregulates Akt Expression in KA-Induced Seizures

Administration of KA triggers status epilepticus and is associated with neuronal apoptosis [22]. In this study, changes in cleaved caspase-3 expression following KA treatment were assessed by Western blotting. Fourteen days after KA treatment, the band intensities of both caspase-3 and cleaved caspase-3 were significantly increased, as shown in Figure 5A. Animals treated with KA alone displayed significantly higher levels of caspase-3 (*p* < 0.001) and cleaved caspase-3 (*p* < 0.05) than control animals. By contrast, pinostrobin treatment significantly reduced the levels of caspase-3 (*p* < 0.001) and cleaved caspase-3 (*p* < 0.05) compared with the KA-alone group (Figure 5D,E).

Protein kinase B (Akt) plays a critical role in neuronal survival following KA-induced seizures [23]. As shown in Figure 5A,B, Akt expression was significantly lower in the KA-alone group than in the control group (*p* < 0.05). Notably, pinostrobin treatment significantly increased Akt and p-Akt expression (*p* < 0.05 and *p* < 0.001, respectively) compared with the KA-alone group (Figure 5B,C). These findings suggest that the neuroprotective effects of pinostrobin against KA-induced neuronal cell loss may be associated with increased Akt expression and reduced cleaved caspase-3 expression.

## 3. Discussion

Epilepsy is a neurological disorder characterised by recurrent, spontaneous seizures. Numerous studies have demonstrated that prolonged seizures lead to apoptotic neuronal death within the hippocampus [24,25]. In this study, i.c.v. injection of KA produced seizures classified as grades 4–5 on the Racine scale, accompanied by neuronal cell loss in the CA1, CA3, and hilar regions of the hippocampus. Notably, administration of pinostrobin (40 mg/kg) significantly attenuated KA-induced neuronal loss in the CA1 and CA3 regions and preserved hilar neurons, as indicated by the maintenance of neuronal density. In models of temporal lobe epilepsy, transplantation of GABAergic progenitor interneurons has been shown to reduce seizure activity and enhance the survival of adult-born neurons in epileptic mice [26]. Therefore, pinostrobin-mediated preservation of hilar neurons may contribute to protection against KA-induced excitotoxicity.

Accumulating evidence indicates that KA administration produces severe seizure activity accompanied by hippocampal neuronal loss and glial responses involving both astrocytes and microglia [15,19,20]. In the present study, KA induced reactive astrocytosis, while pinostrobin treatment further increased GFAP immunoreactivity within the hippocampal CA1, CA3, and hilar regions. Astrocyte activation has been shown to play an important role in regulating brain glucose metabolic homeostasis and has been associated with the prevention of hippocampal neuronal loss and increased GABA levels [18]. Moreover, GABA release from astrocytes plays a key role in inhibiting generalised epilepsy characterised by spontaneous spike-and-wave discharges [27,28]. KA injection also triggers a rapid microglial response in the hippocampus following excitotoxic neuronal damage and seizure activity [29,30]. Activated microglia promote the production of pro-inflammatory cytokines, contributing to hippocampal cell death and prolonged seizure activity [31]. In this study, the KA-induced increase in Iba1-positive microglia was significantly reduced by pinostrobin, suggesting that modulation of the microglial response may contribute, at least in part, to its neuroprotective effects.

KA induces epileptic seizures and excitotoxic neuronal cell death in the hippocampus. Previous studies have demonstrated that KA administration markedly increases levels of the pro-apoptotic protein Bax, whereas levels of the anti-apoptotic protein Bcl-2 decrease following KA treatment. The accumulation of Bax and downregulation of Bcl-2 at the mitochondrial membrane contribute to KA-induced excitotoxic hippocampal cell death [5,6,32]. Pinostrobin has been shown to suppress DNA fragmentation and decrease the Bax/Bcl-2 ratio in a model of β-amyloid-induced neurotoxicity in pheochromocytoma (PC12) cells [11]. Moreover, pinostrobin downregulates the apoptotic markers caspase-3 and Bax while upregulating the anti-apoptotic marker Bcl-2 in a neurotoxin-induced model of Parkinson’s disease, thereby protecting dopaminergic neurons in zebrafish [12]. In the present study, Western blot analysis demonstrated that pinostrobin treatment significantly decreased Bax levels while increasing Bcl-2 expression. The resulting reduction in the pro-apoptotic-to-anti-apoptotic protein ratio (Bax/Bcl-2 ratio) may contribute to protection against KA-induced hippocampal cell loss. Additionally, caspase-3 interacts with several components of apoptotic cascades, including members of the Bcl-2 family and poly(ADP-ribose) polymerase (PARP). Activated caspase-3 cleaves PARP, thereby facilitating apoptotic signalling in response to KA-induced excitotoxicity [33,34]. Recent evidence suggests that KA administration leads to caspase-3 activation, which is associated with prolonged seizure activity and neuronal cell death in the hippocampus [15,35]. KA administration has also been shown to downregulate Akt, contributing to hippocampal neuronal death [36,37]. In the current study, pinostrobin treatment increased Akt expression while decreasing caspase-3 expression, consistent with an anti-apoptotic effect and enhanced hippocampal neuronal survival following KA-induced injury.

Although the present study did not include a pinostrobin-only group, existing in vivo evidence indicates that pinostrobin has a favourable safety profile under physiological conditions. Previous toxicological study of pinostrobin has reported no evidence of systemic toxicity, treatment-related behavioural alterations, or histopathological damage in healthy rodents, supporting its safety in the absence of pathological insults [14].

Behavioural and cognitive evaluations were not conducted in the present study; however, the histological and molecular findings provide evidence of the neuroprotective effects of pinostrobin in the hippocampus. KA-induced excitotoxicity causes extensive hippocampal neuronal degeneration [3]. In the present study, pinostrobin attenuated apoptosis-related signalling and enhanced neuronal survival in the hippocampus. These findings are consistent with previous reports of the antioxidant and neuroprotective properties of pinostrobin [38]. Previous studies have also reported beneficial effects of pinostrobin on cognitive impairment in other experimental models [39,40]. However, whether the neuroprotective effects observed here translate into functional improvements cannot be determined from the present study. Future studies incorporating behavioural and cognitive assessments will be needed to determine the functional consequences of pinostrobin treatment.

Several limitations of the present study should be acknowledged. First, all pathological and molecular assessments were performed on day 14 after KA exposure. Therefore, the study cannot establish a temporal relationship between the observed molecular changes and neuronal protection, as these changes may represent either mechanisms underlying neuroprotection or consequences of reduced neuronal injury. Future studies incorporating analyses at earlier time points are warranted to clarify the temporal sequence of these events. Second, seizure severity, duration, and burden were not quantitatively assessed, and electroencephalogram (EEG) monitoring was not performed. Therefore, it cannot be determined whether the observed neuroprotective effects resulted solely from direct effects on neuronal survival or were partly attributable to attenuation of KA-induced seizure activity. Because pinostrobin was administered both before and after KA exposure, a potential antiseizure effect during the initial seizure episode cannot be excluded. Future studies incorporating quantitative seizure monitoring and EEG recordings are warranted to clarify the relative contributions of antiseizure and neuroprotective effects. Third, the absence of a pinostrobin-only control group limits the ability to evaluate the effects of pinostrobin in healthy, non-epileptic animals. In addition, the absence of a positive-control group treated with a standard antiepileptic or neuroprotective drug prevents direct comparison with an established therapeutic agent. Future studies incorporating appropriate control groups would provide a stronger basis for evaluating the effects of pinostrobin. Fourth, the absence of phosphatase inhibitors may have influenced the assessment of phosphorylated proteins. In addition, the involvement of the Akt and Bax/Bcl-2/caspase-3 signalling pathways was evaluated based on changes in protein expression without pathway-specific inhibition or other mechanistic manipulation. Therefore, the present findings demonstrate an association but do not establish a causal role for these pathways in the neuroprotective effects of pinostrobin. Furthermore, other potentially relevant signalling pathways were not investigated. Future studies using pathway-specific inhibition or manipulation are warranted to further clarify the underlying mechanisms. Fifth, GABAergic interneurons were not specifically identified using GABAergic neuronal markers. Although Nissl staining allowed the assessment of morphologically preserved neurons in the hippocampal hilus, it cannot distinguish GABAergic interneurons from other neuronal populations, such as excitatory mossy cells. Future studies using specific markers, such as glutamate decarboxylase 67 (GAD67), are warranted to confirm the effects of pinostrobin on hilar GABAergic interneurons. Finally, an a priori power analysis was not performed to determine the required sample size, and formal statistical tests for normality and outliers were not conducted. No data points were excluded based on statistical outlier criteria. Future studies with an appropriately justified sample size and formal assessment of statistical assumptions are warranted to provide stronger statistical support for the findings.

## 4. Materials and Methods

### 4.1. Animals and Study Design

Fifteen male Wistar rats (8 weeks old, weighing 250–270 g) were obtained from Nomura Siam International Co., Ltd. (Bangkok, Thailand). The animals were maintained under controlled conditions (12-h light–dark cycle, 25 °C ± 2 °C, 50–60% humidity), with ad libitum access to food and water.

All animal experimental protocols were conducted in accordance with the Ethical Principles and Guidelines for the Use of Animals for Scientific Purposes of the National Research Council of Thailand. The study was approved by the Animal Ethics Committee of the University of Phayao, Thailand (approval no. 1-027-67).

The animals were randomly assigned to three experimental groups (*n* = 5 per group): the control group, KA group, and pinostrobin 40 mg/kg group. Pinostrobin was formulated, and the dose used in this study was selected based on our previous reports [10,41]. Animals in the pinostrobin group received pinostrobin at a dose of 40 mg/kg (dissolved in 0.5% carboxymethylcellulose sodium [CMCS]; Sigma-Aldrich, St. Louis, MO, USA) once daily by oral gavage for 7 days before i.c.v. injection of KA. Pinostrobin treatment was continued for an additional 14 days after KA injection. Control and KA-alone animals received an equivalent volume of CMCS vehicle.

### 4.2. i.c.v. Injection of Kainic Acid

The animals were anaesthetised by intraperitoneal (i.p.) injection of thiopental sodium (50 mg/kg). An adequate depth of anaesthesia was confirmed by the absence of a withdrawal response to a pin-prick stimulus applied to the hind paw. The animals were then placed in a stereotactic apparatus (RWD Life Science Co., Ltd., Shenzhen, China). Coordinates relative to bregma were selected to target the lateral ventricle (−0.8 mm AP, −1.4 mm ML, and −3.3 mm DV). KA (cat. no. FK46986; Biosynth Ltd., Compton, UK) was dissolved in sterile normal saline (1 mg/mL). A KA volume of 1 µL (1000 ng) was selected based on a previous study [42] and administered using a Hamilton syringe over 3 min. The cannula was kept in place for an additional 3 min to prevent backflow and was then slowly withdrawn from the brain. Control animals received an equivalent volume of sterile normal saline.

Following the procedure, KA-treated animals exhibited seizure behaviours, including forelimb clonus, rearing, and generalised tonic–clonic seizures (stages 3–5 according to the Racine scale [43]). All saline-injected animals showed no seizure behaviours (stage 0 according to the Racine scale). Specific humane endpoints were established to minimise animal suffering. All animals were monitored continuously for the first 6 h after injection and daily thereafter until the end of the experiment. Animals were humanely euthanised if they met either of the following criteria: continuous generalised tonic–clonic seizures (Racine scale stage 5) lasting more than 90 min or body weight loss exceeding 20% relative to baseline. Animals meeting these criteria were immediately euthanised with thiopental sodium (70 mg/kg, i.p.). No animals died before reaching these endpoints.

### 4.3. Nissl Staining and Immunohistochemistry Staining for Astrocytes and Microglia

At the end of the experiment, all animals were euthanised with thiopental sodium (70 mg/kg, i.p.) and then transcardially perfused with ice-cold sterile normal saline. The brains were removed, and the left hemispheres were fixed in 4% paraformaldehyde in phosphate-buffered saline (PBS) (0.1 M, pH 7.4) for 48 h. The tissues were subsequently cryoprotected in 30% sucrose in PBS for an additional 48 h and then cut into 30-µm-thick coronal sections using a cryostat (CM1950; Leica Microsystems, Inc., Wetzlar, Germany). The sections were mounted on positively charged slides, and Nissl staining with cresyl violet dye (cat. no. 1.05235; Sigma-Aldrich) was performed to visualise hippocampal neurons.

For immunohistochemical staining, free-floating sections were incubated in blocking solution (10% horse serum and 0.1% Triton X-100 in PBS) for 90 min. Primary antibodies against GFAP (1:300, cat. no. MAB5628; Sigma-Aldrich; Merck KGaA, Darmstadt, Germany) or Iba1 (1:250, cat. no. MABN92; Sigma-Aldrich; Merck KGaA, Darmstadt, Germany) were applied overnight at 4 °C. The sections were subsequently incubated with secondary antibody (1:500, cat. no. 715-065-150; Jackson ImmunoResearch, West Grove, PA, USA) for 2 h at room temperature, followed by signal amplification with ExtrAvidin peroxidase (1:1000, cat. no. E2886; Sigma-Aldrich) for 1 h. Finally, the signal was developed using 3,3′-diaminobenzidine as the chromogen.

Cell quantification was performed on sections of the dorsal hippocampus at stereotaxic coordinates 3.8–4.0 mm posterior to bregma. Images from every sixth section were captured using an Eclipse Ni-U upright microscope (Nikon, Tokyo, Japan) and analysed with NIS-Elements imaging software version 5 (Nikon, Tokyo, Japan). For Nissl staining, quantification was restricted to mature, post-mitotic neurons identified by characteristic neuronal morphology, including a large euchromatic nucleus and distinct nucleolus. Glial cells, including astrocytes and microglia, were excluded from the counts to allow accurate assessment of neuronal survival. For quantitative analysis, one anatomically matched coronal section at the dorsal hippocampal level was selected from every sixth section. Cell counting was performed within a standardised area of interest measuring 250 µm in length and covering the full thickness of the pyramidal cell layer in the CA1 and CA3 subregions, while the entire hilar region of the dentate gyrus was analysed. Two non-overlapping microscopic fields were evaluated in each of the CA1 and CA3 subregions. The mean value obtained for each animal was used for subsequent statistical analysis. All neuronal counts were performed by an investigator blinded to the experimental groups to minimise bias.

For quantification of GFAP immunoreactivity and Iba1-positive microglia, cells were counted manually by an observer blinded to the experimental groups rather than using automated thresholding software. An individual GFAP-positive astrocyte was identified based on a clearly discernible GFAP-positive cell body(soma). Cells without a clearly identifiable soma, only partially stained cells, and overlapping profiles that could not be reliably assigned to an individual cell were excluded from the count. For quantitative evaluation, one anatomically matched coronal section at the dorsal hippocampal level was selected from every sixth section for analysis. To ensure consistent sampling, cell counting was restricted to a standardised area of interest measuring 250 µm in length and covering the entire thickness of the pyramidal cell layer in the CA1 and CA3 subregions, while the entire hilar region was assessed separately. Two non-overlapping microscopic fields were analysed in each of the CA1 and CA3 subregions. The mean glial cell count for each animal was used for statistical analysis. All results were expressed as a percentage of the control (% of control).

### 4.4. Western Blotting

Right hippocampal tissue from four animals per group was used for Western blot analysis due to the limitation in the number of available lanes per gel. Four animals per group were selected to allow consistent sample loading and experimental conditions across groups and to ensure the reliability and reproducibility of each Western blot run. The tissue was homogenised in RIPA lysis buffer (cat. no. RB4475; BioBasic, Markham, ON, Canada) supplemented with 1% protease inhibitor cocktail set I (cat. no. 539131; Sigma-Aldrich, St. Louis, MO, USA). Lysates were clarified by centrifugation at 10,000× *g* for 15 min at 4 °C, and protein concentrations were determined using a Qubit Protein Assay Kit (cat. no. Q33211; Thermo Fisher Scientific Inc., Waltham, MA, USA) and Qubit 4 Fluorometer (Invitrogen, Waltham, MA, USA) according to the manufacturers’ protocols. Equal amounts of protein (30 µg) were separated by 10% sodium dodecyl sulphate-polyacrylamide gel electrophoresis and transferred to polyvinylidene difluoride membranes (cat. no. IPVH00010; Merck Millipore, Burlington, MA, USA). Samples from all experimental groups were processed and transferred under identical conditions on the same blot. After transfer, each membrane was cut according to the molecular weights of the target proteins, allowing the same membrane to be used to probe the proteins of interest and the β-actin loading control for normalisation. The membranes were blocked with 5% skimmed milk in Tris-buffered saline containing 0.1% Tween-20 (TBST) and incubated overnight at 4 °C with the appropriate primary antibodies: anti-cleaved caspase-3 (1:1000, cat. no. AF7022; Affinity Biosciences, Cincinnati, OH, USA), anti-caspase-3 (1:1000, cat. no. E-AB-13815; Elabscience, Houston, TX, USA), anti-pan-Akt1/2/3 (1:1000, cat. no. AF6261; Affinity Biosciences, Cincinnati, OH, USA), anti-phospho-Akt1/2/3 (1:1000, cat. no. AF0016; Affinity Biosciences, Cincinnati, OH, USA), anti-Bcl-2 (1:1000, cat. no. AF6139; Affinity Biosciences, Cincinnati, OH, USA), anti-Bax (1:1000, cat. no. ES1753; ELK Biotechnology, Wuhan, China), and anti-β-actin (1:2000, cat. no. E-AB-40517; Elabscience Biotechnology Co., Ltd., Wuhan, China). The membranes were washed with TBST and incubated with horseradish peroxidase-conjugated goat anti-rabbit secondary antibody (1:5000, cat. no. AP307P; Sigma-Aldrich, St. Louis, MO, USA) for 2 h at room temperature. Immunoreactive bands were visualised using an enhanced chemiluminescence kit and captured with a chemiPRO XS Western Blot Imaging System (Cleaver Scientific, Rugby, UK). Protein band densities were quantified using ImageJ software (version 1.54d; National Institutes of Health, Bethesda, MD, USA) and normalised to β-actin.

### 4.5. Statistical Analysis

Data were analysed using one-way analysis of variance followed by Tukey’s multiple-comparisons test in GraphPad Prism version 9.0. A *p* value of <0.05 was considered statistically significant.

## 5. Conclusions

In summary, pinostrobin treatment was associated with reduced hippocampal neuronal loss and changes in glial responses and apoptosis-related markers following KA exposure, including decreased levels of Bax and caspase-3 and increased levels of Bcl-2 and Akt. Furthermore, pinostrobin reduced the number of Iba1-positive microglia and increased GFAP immunoreactivity following KA-induced injury. Together, these findings suggest that pinostrobin may have neuroprotective effects in KA-induced hippocampal injury. However, the underlying mechanisms and the contribution of these effects to seizure severity and KA-induced epileptogenesis remain to be established.

## Figures and Tables

**Figure 1:**
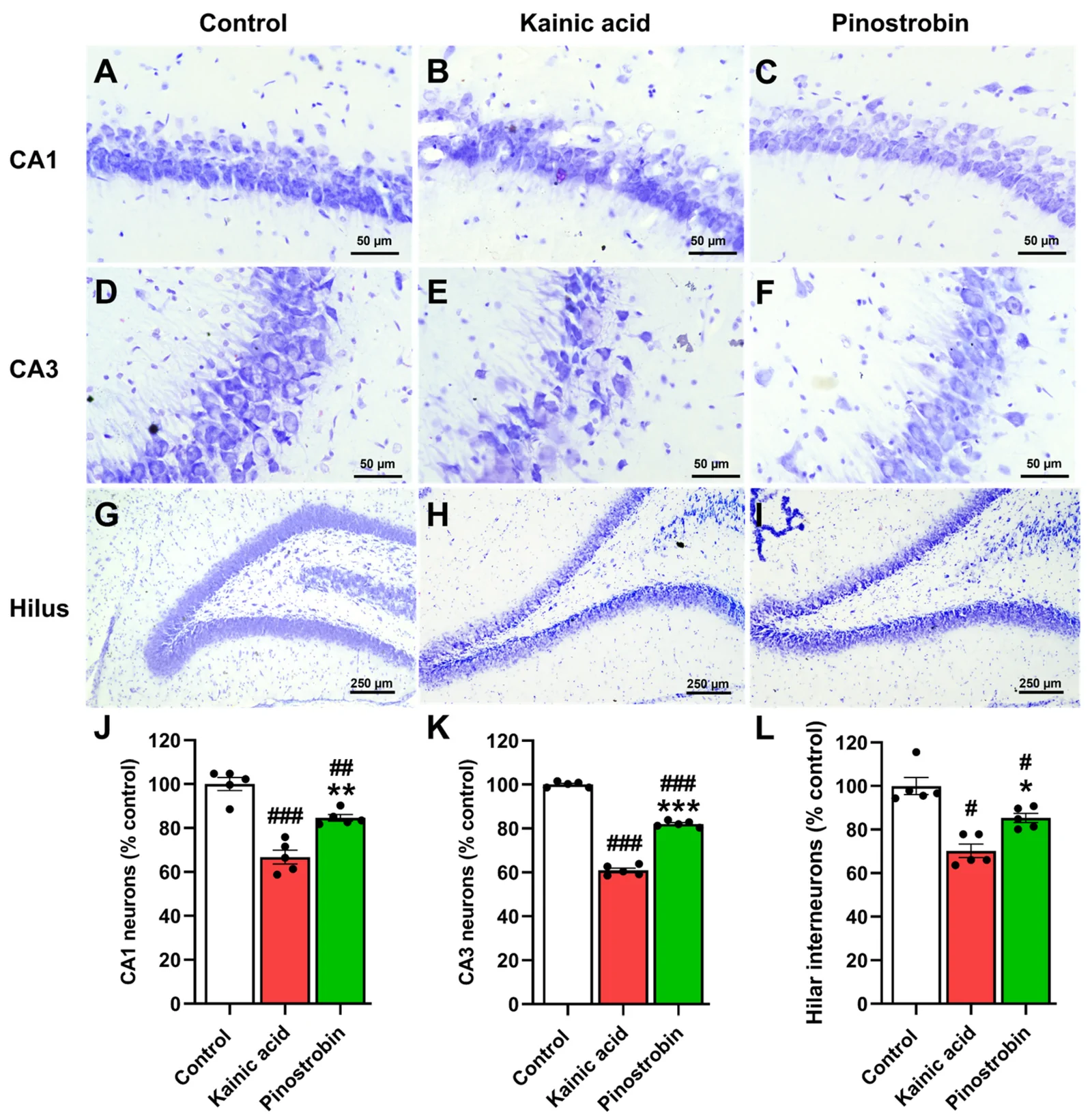
Pinostrobin attenuates KA-induced neuronal loss in the hippocampal subfields. Nissl staining was performed to assess neuronal cell survival in the CA1 (**A**–**C**), CA3 (**D**–**F**), and hilus (**G**–**I**) on day 14 after KA injection. Neuronal density was quantified in the CA1 (**J**), CA3 (**K**), and hilus (**L**), and expressed as a percentage of the control. Scale bars: (**A**–**F**) = 50 µm; (**G**–**I**) = 250 µm. Data are presented as the mean ± SEM (*n* = 5 per group). # *p* < 0.05, ## *p* < 0.01, and ### *p* < 0.001 versus the control group; * *p* < 0.05, ** *p* < 0.01, and *** *p* < 0.001 versus the KA group.

**Figure 2:**
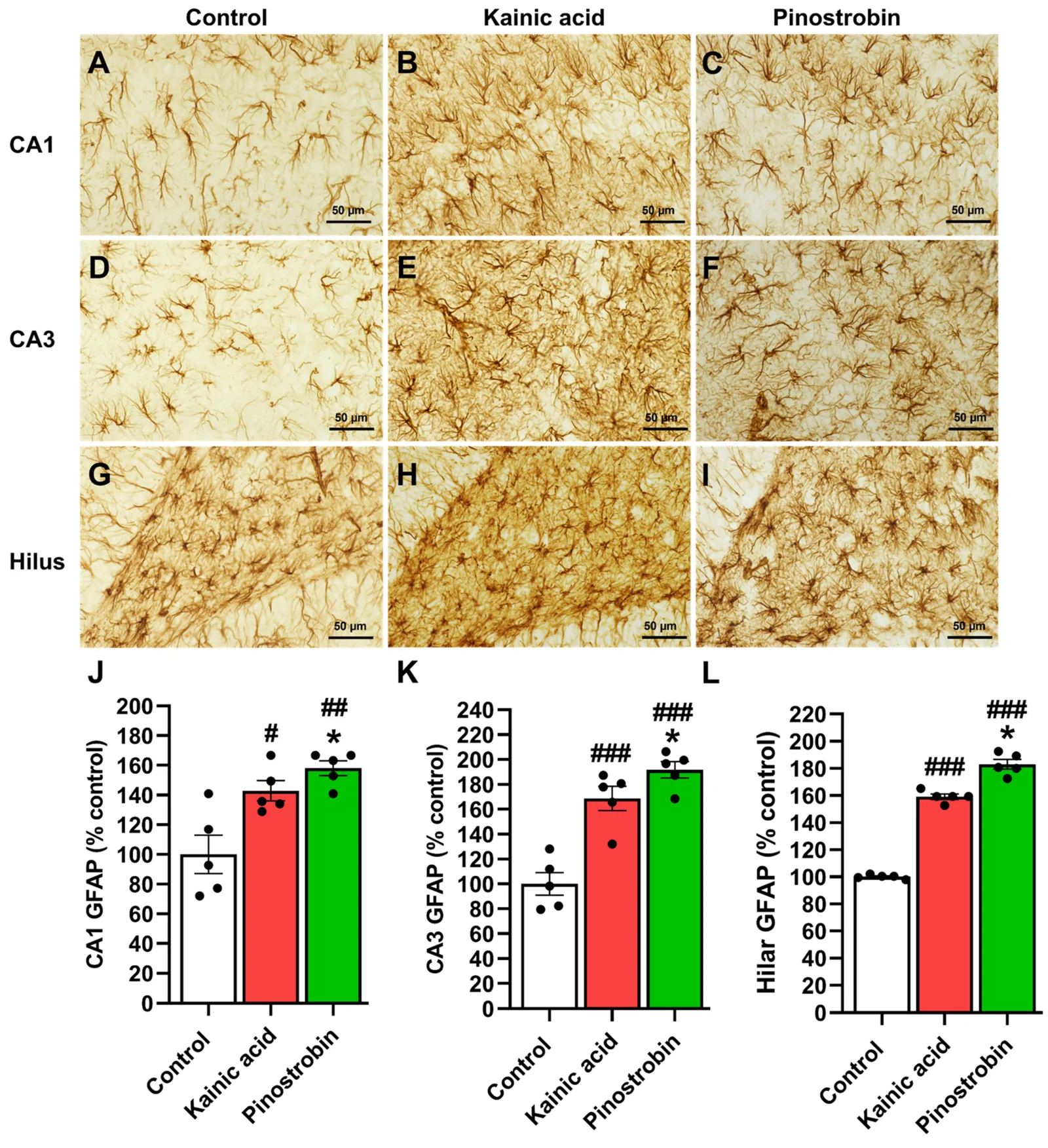
Pinostrobin increases GFAP immunoreactivity following KA-induced neuronal cell loss in the hippocampal subfields. GFAP immunoreactivity was assessed in the CA1 (**A**–**C**), CA3 (**D**–**F**), and hilus (**G**–**I**) on day 14 after KA injection. GFAP immunoreactivity was quantified in the CA1 (**J**), CA3 (**K**), and hilus (**L**) and expressed as a percentage of the control. Scale bar = 50 µm. Data are presented as the mean ± SEM (*n* = 5 per group). # *p* < 0.05, ## *p* < 0.01, and ### *p* < 0.001 versus the control group; * *p* < 0.05 versus the KA group.

**Figure 3:**
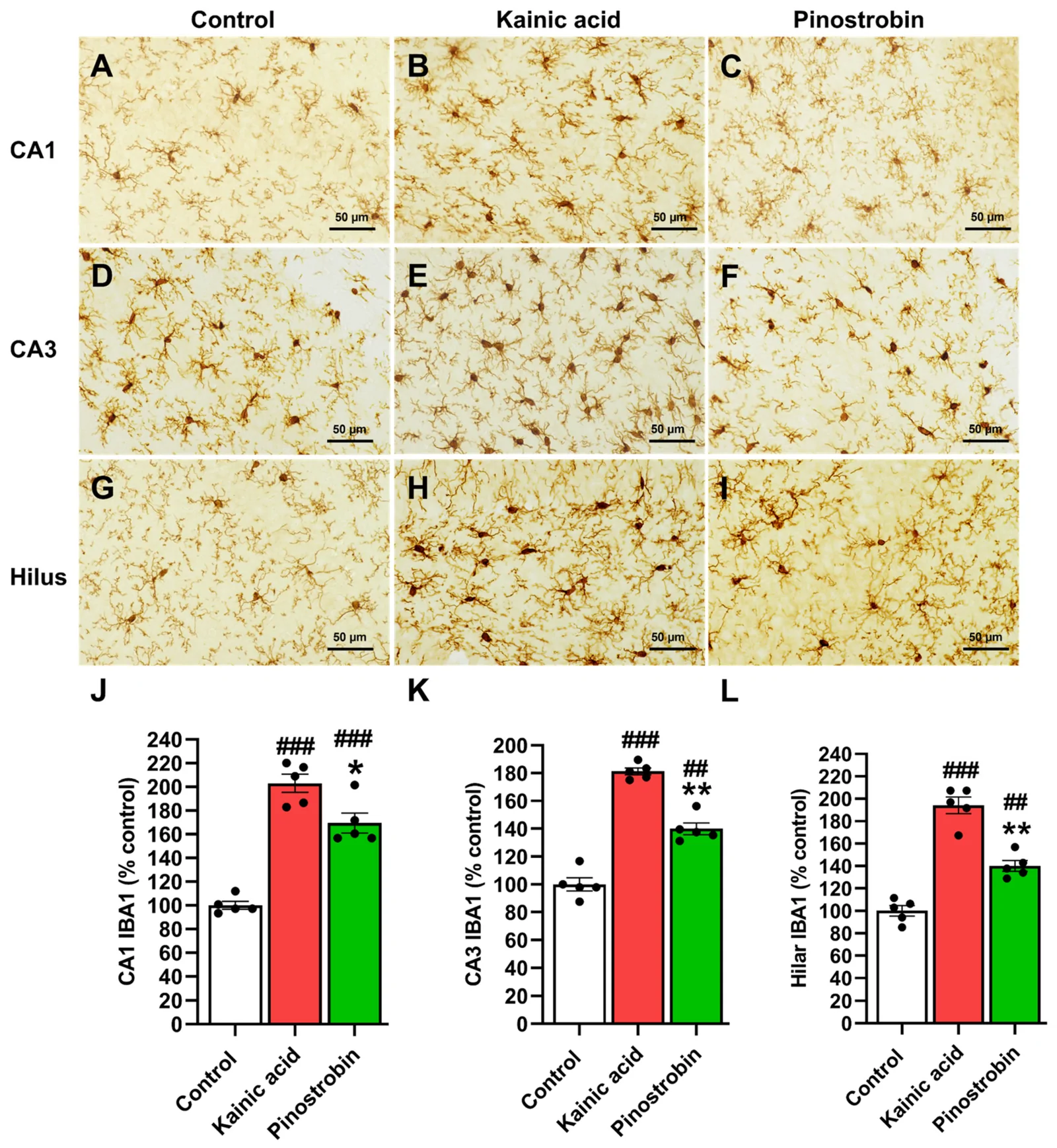
Pinostrobin reduces the number of Iba1-positive microglia following KA-induced neuronal cell loss in the hippocampus. Iba1 immunoreactivity was assessed in the CA1 (**A**–**C**), CA3 (**D**–**F**), and hilus (**G**–**I**) on day 14 after KA injection. Iba1-positive microglia were quantified in the CA1 (**J**), CA3 (**K**), and hilus (**L**) and expressed as a percentage of the control. Scale bar = 50 µm. Data are presented as the mean ± SEM (*n* = 5 per group). ## *p* < 0.01 and ### *p* < 0.001 versus the control group; * *p* < 0.05 and ** *p* < 0.01 versus the KA group.

**Figure 4:**
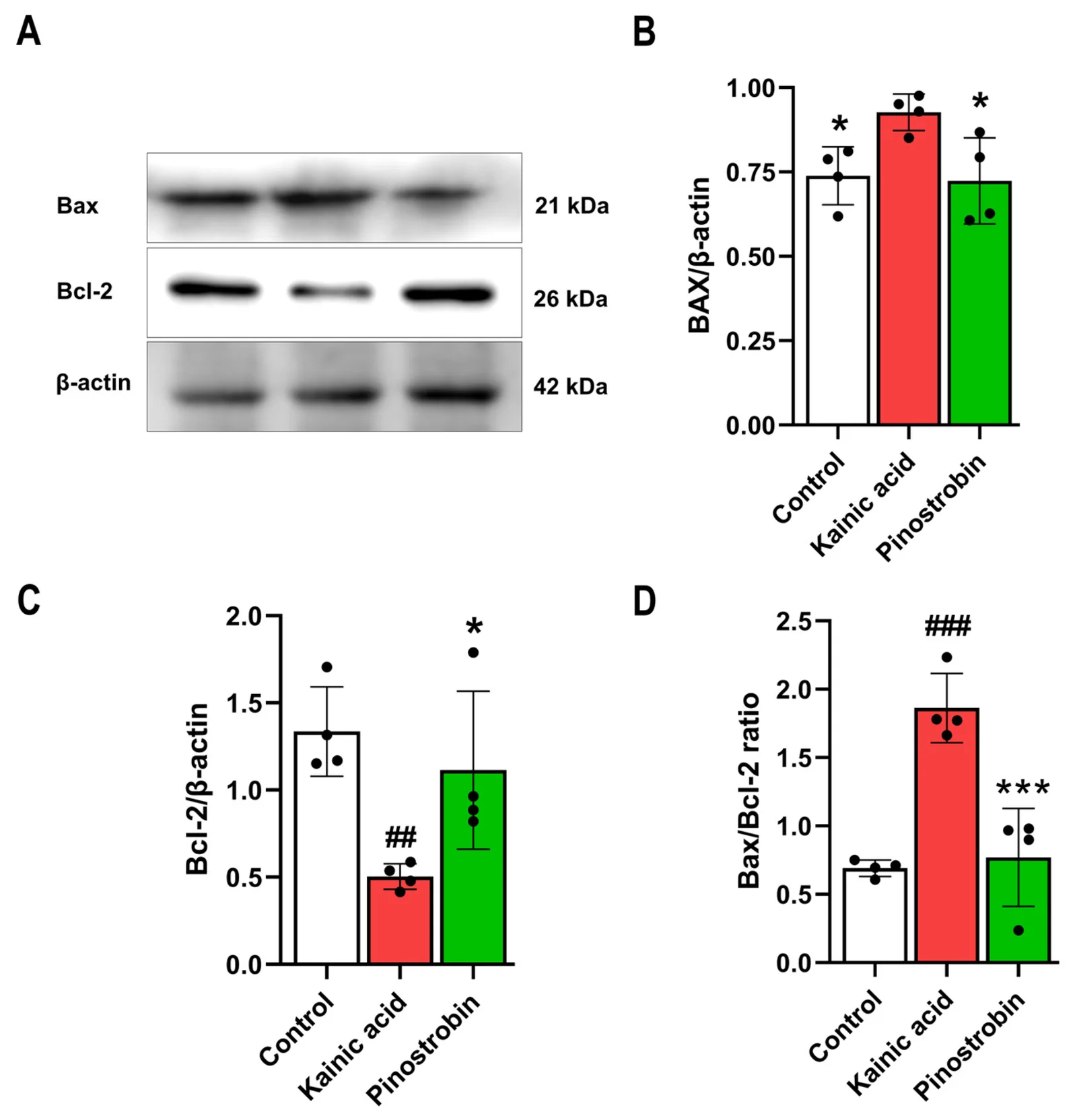
Pinostrobin modulates the expression of apoptosis-related proteins in the hippocampus on day 14 after KA injection. Representative immunoblots showing protein bands for Bax, Bcl-2, and β-actin (**A**). Quantitative analysis of Bax (**B**) and Bcl-2 (**C**) levels and the Bax/Bcl-2 ratio (**D**), as determined by Western blotting. Data are presented as the mean ± SD (*n* = 4 per group). ## *p* < 0.01 and ### *p* < 0.001 versus the control group; * *p* < 0.05 and *** *p* < 0.001 versus the KA group.

**Figure 5:**
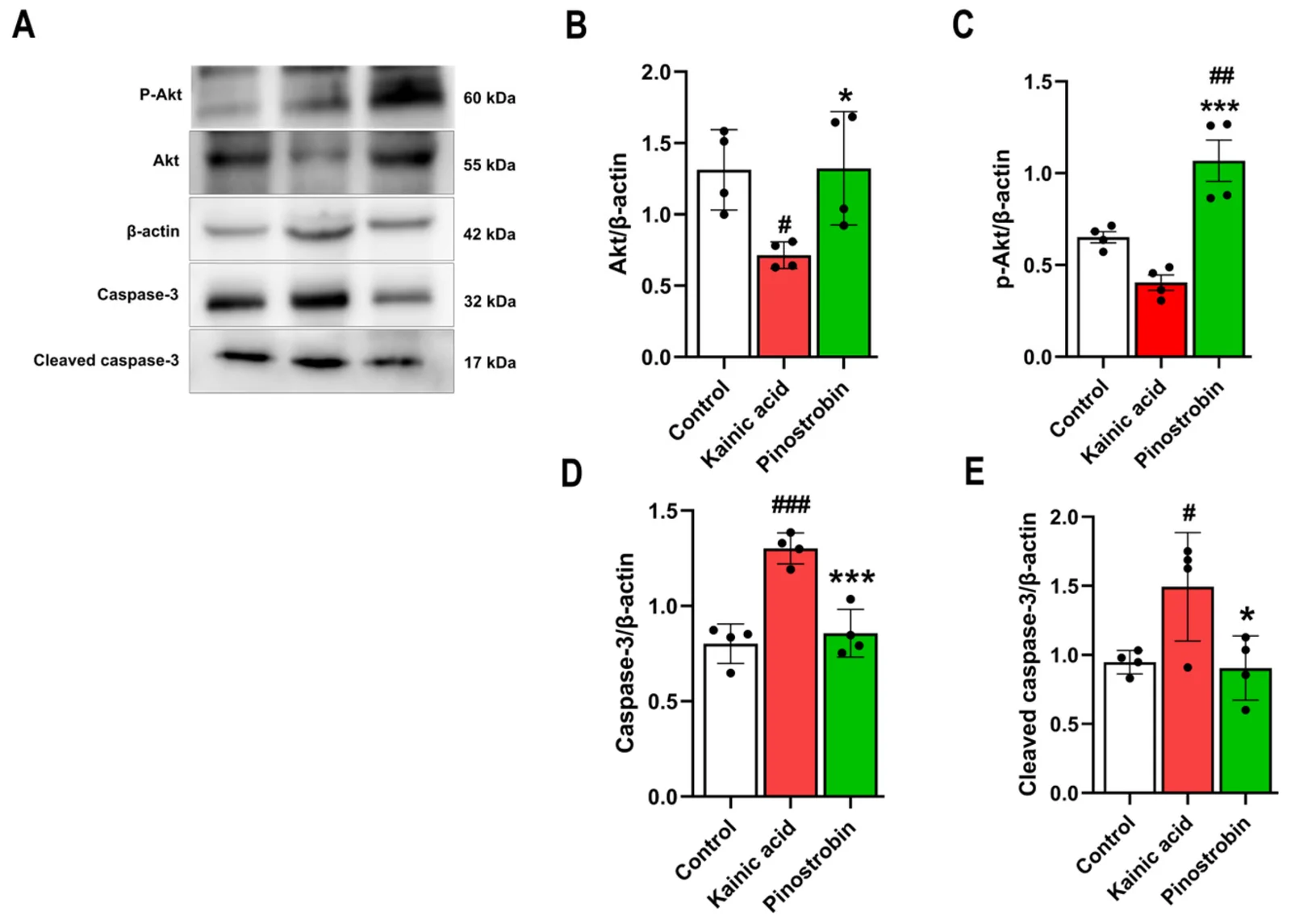
Pinostrobin increases Akt expression while reducing caspase-3 and cleaved caspase-3 expression in the hippocampus on day 14 after KA injection. Representative immunoblots showing protein bands for p-Akt, Akt, caspase-3, cleaved caspase-3, and β-actin (**A**). Quantitative analysis of Akt (**B**), p-Akt (**C**), caspase-3 (**D**), and cleaved caspase-3 (**E**) levels normalised to β-actin. Data are presented as the mean ± SD (*n* = 4 per group). # *p* < 0.05, ## *p* < 0.01 and ### *p* < 0.001 versus the control group; * *p* < 0.05 and *** *p* < 0.001 versus the KA group.

## Data Availability

The original contributions presented in this study are included in the article. Further inquiries can be directed to the corresponding author.
